# Cardiovascular autonomic regulation correlates with cognitive performance in patients with a history of traumatic brain injury

**DOI:** 10.1007/s10072-023-06857-y

**Published:** 2023-05-25

**Authors:** Ruihao Wang, Dafin Muresanu, Katharina Hösl, Max J. Hilz

**Affiliations:** 1 Department of Neurology, University of Erlangen-Nuremberg, Schwabachanlage 6, 91054 Erlangen, Germany; 2 Department of Neurosciences, Iuliu Hatieganu University of Medicine and Pharmacy, Cluj-Napoca-Napoca, Romania; 3 RoNeuro Institute for Neurological Research and Diagnostic, Cluj-Napoca-Napoca, Romania; 4 Department of Psychiatry and Psychotherapy, Paracelsus Medical University, Nuremberg, Germany; 5 Department of Neurology, Icahn School of Medicine at Mount Sinai, New York, NY USA

**Keywords:** Cardiovascular autonomic regulation, Cognitive performance, Executive function, Visuospatial function, Traumatic brain injury, Correlations

## Abstract

**Background and objective:**

Traumatic brain injury (TBI) may afflict brain areas contributing to both cardiovascular autonomic regulation and cognitive performance. To evaluate possible associations between both functions in patients with a history of TBI (post-TBI-patients), we determined correlations between cardiovascular autonomic regulation and cognitive function in post-TBI-patients.

**Methods:**

In 86 post-TBI-patients (33.1 ± 10.8 years old, 22 women, 36.8 ± 28.9 months after injury), we monitored RR intervals (RRI), systolic and diastolic blood pressures (BPsys, BPdia), and respiration (RESP) at rest. We calculated parameters of total cardiovascular autonomic modulation (RRI-standard-deviation (RRI-SD), RRI-coefficient-of-variation (RRI-CV), RRI-total-powers), sympathetic (RRI-low-frequency-powers (RRI-LF), normalized (nu) RRI-LF-powers, BPsys-LF-powers) and parasympathetic modulation (root-mean-square-of-successive-RRI-differences (RMSSD), RRI-high-frequency-powers (RRI-HF), RRI-HFnu-powers), sympathetic-parasympathetic balance (RRI-LF/HF-ratios), and baroreflex sensitivity (BRS). We used the Mini-Mental State Examination and Clock Drawing Test (CDT) to screen the general global and visuospatial cognitive function, and applied the standardized Trail Making Test (TMT)-A assessing visuospatial abilities and TMT-B assessing executive function. We calculated correlations between autonomic and cognitive parameters (Spearman’s rank correlation test; significance: *P* < 0.05).

**Results:**

CDT values positively correlated with age (*P* = 0.013). TMT-A values inversely correlated with RRI-HF-powers (*P* = 0.033) and BRS (*P* = 0.043), TMT-B values positively correlated with RRI-LFnu-powers (*P* = 0.015), RRI-LF/HF-ratios (*P* = 0.036), and BPsys-LF-powers (*P* = 0.030), but negatively with RRI-HFnu-powers (*P* = 0.015).

**Conclusions:**

In patients with a history of TBI, there is an association between decreased visuospatial and executive cognitive performance and reduced parasympathetic cardiac modulation and baroreflex sensitivity with relatively increased sympathetic activity. Altered autonomic control bears an increased cardiovascular risk; cognitive impairment compromises quality of life and living conditions. Thus, both functions should be monitored in post-TBI-patients.

**Supplementary Information:**

The online version contains supplementary material available at 10.1007/s10072-023-06857-y.

## Introduction

Cognitive impairment is among the common long-term sequelae of traumatic brain injury (TBI) [1]. Particularly visuospatial and executive functions are often compromised in patients with a history of TBI (post-TBI-patients) [2].

Moreover, there is growing evidence that post-TBI-patients may have subtle cardiovascular autonomic dysfunction even a long time after the injury [3–7]. In several studies of patients with a history of mild to severe TBI, our group found subtle cardiovascular autonomic dysfunction, including an overall decrease in cardiovascular autonomic modulation with relative sympathetic predominance already at rest and altered baroreflex sensitivity with inadequate autonomic adjustment to baroreflex loading or unloading [3–5]. Moreover, baroreflex-independent activation of the oculocardiac reflex triggered paradox sympathetic instead of parasympathetic cardiovascular responses even years after patients had experienced mild, moderate, or severe TBI [6, 7].

To our knowledge, there are no studies so far that assessed associations between altered autonomic and cognitive functions in patients with a history of TBI. However, several studies demonstrated correlations between cognitive performance and cardiovascular autonomic regulation in healthy persons [8], physically disabled women aged 65 and older [9], elderly persons with cardiovascular risk factors [10], and patients with hypertension [11], Alzheimer’s disease [12, 13], or chronic fatigue syndrome [14]. Given these associations and the fact that TBI-related brain lesions may disrupt neural structures that are responsible for both cardiovascular autonomic regulation and cognitive function [15], such as the prefrontal cortex (PFC), anterior cingulate cortex, subcortical structures like insula, amygdala, and the cortico-subcortical circuits [16], we hypothesize that there are associations between cardiovascular autonomic dysfunction and cognitive impairment in patients with a history of TBI.

In this study, we therefore aimed to evaluate whether there are correlations between cardiovascular autonomic function and cognitive performances in post-TBI-patients.

## Patients and methods

### Study patients

In patients who had experienced a mild, moderate, or severe TBI, we performed cardiovascular autonomic testing and assessed cognitive function. The diagnosis of mild TBI was established according to WHO operational criteria [17] including [1] one or more of the following: confusion or disorientation; loss of consciousness for 30 min or less; post-traumatic amnesia for less than 24 h; and/or other transient neurological abnormalities such as focal signs, seizure, and intracranial lesion not requiring surgery; and [2] Glasgow Coma Scale (GCS) scores of 13–15 after 30 min post-injury or later upon presentation to health care [3, 4, 7, 17].

To identify patients with a history of TBI, we had to rely on patient records and personal information from patients or relatives. Since we were not evaluating patients who were acutely afflicted by a TBI but studied patients whose initial trauma had occurred months or years prior to our evaluation, we had to avoid any incorrect classification of the initial trauma as moderate or as severe TBI; moreover, we intended to be consistent with our previous studies of autonomic function in patients with a history of moderate-severe TBI [5, 6]. Hence, we did not distinguish between patients with a history of moderate TBI and patients with a history of severe TBI. Instead, we used the Mayo Clinic Classification of TBI severity [18] that assigns the diagnoses of moderate or severe TBI if one or more of the following criteria applied [18]: (1) death due to this TBI; (2) loss of consciousness of more than 30 min; (3) post-traumatic anterograde amnesia of more than 24 h; (4) worst GCS score within the first 24 h of less than 13 (unless, e.g., attributable to intoxication, sedation, systemic shock); (5) more than one of the following was present: intracerebral, subdural, or epidural hematoma, cerebral or hemorrhagic contusion, dura penetration, subarachnoid hemorrhage, brain stem injury [6, 18].

All patients were recruited from the TBI registry of the University of Erlangen-Nuremberg. Selection of patients participating in our study was based on the medical records at the time of the injury. Unless medical records were complete and available for review to confirm injury information, patients were not enrolled in our study. To rule out selection errors due to erroneous trauma recollection, we also excluded patients from the study if records depended on self-reported information only [6, 7].

In addition, we excluded patients from the study in whom the TBI manifestations were due to drugs, alcohol, and medications, caused by other injuries or treatment for other injuries. We also excluded patients with a history of diseases or on any medication possibly affecting the autonomic function.

Moreover, we only included patients who had no clinically overt neurological or autonomic dysfunction, did not complain about any cognitive difficulties, and pursued their daily and professional tasks without overt post-traumatic limitations.

The study was approved by the Ethics Committee of the University of Erlangen-Nuremberg. Prior to the study, all patients had given their written informed consent according to the Declaration of Helsinki.

### Measurements of bio-signals

We tested the patients between 9 a.m. and 2 p.m., after a resting period of at least 40 min that ensured a stable cardiovascular situation. Cardiovascular autonomic testing was performed under resting conditions in a quiet room with an ambient temperature of 24 °C and stable humidity. Study conditions were standardized throughout the entire observational period.

Under resting conditions, we recorded RRIs (ms) by 3-lead electrocardiography, BPsys and BPdia (mmHg) by finger-pulse photoplethysmography (Portapress; TPD-Biomedical Instrumentation, Amsterdam, NL), and respiratory frequency (RESP; min^−1^) by chest impedance measurements [4] for 5 min. We extracted the most stationary and artefact-free 120-s epochs to average values of RRIs, BPsys, BPdia, and RESP and to calculate the autonomic parameters described below. Bio-signal data were digitized and displayed on a personal computer and a custom-designed data acquisition and analysis system (SUEmpathy™, SUESS-Medizintechnik, Germany) and stored for off-line analysis [4].

### Calculation of time-domain and frequency-domain parameters

We calculated autonomic parameters in the time- and frequency-domains [4, 19].

As time-domain parameters, we determined RRI-SD and the coefficient of variation of RRIs (RRI-CV), both reflecting cardiac sympathetic and parasympathetic modulation [4, 19]. Moreover, we calculated the square root of the mean squared differences of successive RRIs (RMSSD), reflecting cardiac parasympathetic modulation [4, 19].

To assess cardiovascular sympathetic and parasympathetic modulation in the frequency-domain, we performed trigonometric regressive spectral analyses (TRS) of RRI and BPsys values sampled during the 120 s at rest. We determined sympathetic and parasympathetic modulation of RRIs and BPsys in the low-frequency (LF; 0.04–0.14 Hz) and high-frequency (HF; 0.15–0.50 Hz) ranges [4, 19].

LF oscillations of RRIs at rest reflect sympathetic outflow and, to an undetermined degree, also parasympathetic modulation; LF oscillations of BP are related to sympathetic outflow only [19]. HF oscillations of RRIs reflect parasympathetic modulation [19], whereas BP fluctuations in the HF range are primarily a mechanical consequence of respiration-induced fluctuations in venous return and cardiac output [4, 19]. The magnitude of LF and HF oscillations was determined as the integral under the power spectral density curves of RRI (ms^2^/Hz) and BPsys (mm Hg^2^/Hz) for the LF and HF bands, and was expressed as LF and HF powers of RRI (ms^2^) and BP (mm Hg^2^) [4].

As an approximation of the total power (TP) of RRI oscillations and an index of overall cardiac autonomic modulation, we calculated the sum of LF and HF powers in the range from 0.04 to 0.5 Hz [4, 19]. Moreover, we also calculated the ratio between RRI oscillations in the LF and HF ranges, and used the LF/HF ratio of RRI as index of the balance between sympathetic and parasympathetic influences on HR modulation [4, 19]. To adjust for inter-individual differences in sympathetic or parasympathetic modulation and to assess relative changes, we normalized LF and HF powers of RRI by calculating percentage values of LF and HF powers in relation to total powers of RRI fluctuation, with RRI-LFnu = [LF/(LF + HF)] × 100%, and RRI-HFnu = [HF/(LF + HF)] × 100% [19].

Finally, we determined baroreflex sensitivity (BRS) using the TRS software which selected pairs of LF and HF oscillations of BPsys and RRI with high coherence [4]. Coherence spans from 0, i.e., no association, to 1, i.e., maximum association. High coherence at a specific frequency, e.g., > 0.7, indicates a stable phase relation — and thus synchronization — between two signals oscillating at this frequency [4]. Then, the sensitivity of the baroreflex loop (ms mmHg^−1^) was derived as gain values from changes in RRIs (ms) in relation to changes in BPsys (mmHg) [4].

### Assessment of cognitive performance

We used the Mini-Mental State Examination (MMSE) as a screening tool to measure global cognitive function [20]. The test consists of 30 tasks and can be scored by summing the points assigned to each successfully completed task. The MMSE measures orientation in time and space, attention and flexibility, short-term memory, language, constructional ability, reading, and writing [20]. MMSE scores below 24 points are suggestive of cognitive impairment [20].

To evaluate the visuospatial function, we used the clocking drawing test. The patients were asked to draw a clock, to put in numbers, and to set the time, for example, to 10 min past eleven [21]. Testing requires approximately 2 to 5 min. Scoring was performed according to the criteria described by Schulman [21]. The Schulman score was determined using a 6-point scale with a score of 1 indicating a perfect result and a score of 6 indicating a very poor result [21].

We assessed executive function using the standardized Trail Making Test (TMT, part A and part B). In TMT-A, the participant was asked to draw a line connecting consecutively circles that are numbered from 1 to 25 and are scattered across a page [22, 23]. In TMT-B, the participant was asked to draw a line connecting alternating numbers and letters that are scattered across a page, i.e., 1–A with 2–B with 3–C, etc. [22, 23]. Scoring is based on the seconds required to complete the test. While TMT-A assesses visuospatial abilities [24], TMT-B assesses executive function [24, 25], with higher TMT values indicating poorer cognitive function [22, 24].

### Statistical analysis

Data were tested for normal distribution using the Shapiro–Wilk test. We used the Mann–Whitney *U*-test for not normally distributed data and the* t*-test for independent samples in case of normally distributed data to assess whether cardiovascular autonomic parameters and cognitive parameters differed significantly between male and female patients, or between patients with a history of mild TBI and patients with a history of moderate-severe TBI. In all our 86 post-TBI-patients, we correlated the cognitive parameters with age, time duration since the injury, and GCS scores, and with cardiovascular autonomic parameters, using Spearman’s rank correlation test for not normally distributed data and Pearson’s test for normally distributed data. Using the same tests, we calculated correlations between cardiovascular autonomic parameters and values of cognitive tests separately for the 64 male and 22 female post-TBI-patients. Significance was set at *P* < 0.05. For data analysis, we used a commercially available statistical program (IBM SPSS Statistics for Windows, Version 20.0. Armonk, NY, USA).

## Results

Eighty-six TBI patients, 22 women and 64 men, participated in the study. Their mean age was 33.1 ± 10.8 years; the median interval between the TBI and our measurements was 25.5 months (interquartile range 14–55 months). Forty-three patients had experienced a mild TBI, and another 43 patients had suffered a moderate or severe TBI. None of our patients had MMSE scores below 24. MMSE scores did not correlate with age, time since injury, GCS scores, RRIs, BP values, respiration, or time- and frequency-domain autonomic parameters (Tables 1, 2, and 3).

**Table 1:** Correlations between values of MMSE, CDT, TMT-A, and TMT-B and demographic data in 86 patients with a history of traumatic brain injury (TBI)

	MMSE		CDT		TMT-A (s)		TMT-B (s)	
	Rho	*P*	Rho	*P*	Rho	*P*	Rho	*P*
Age (year)	− 0.050	0.645	***0.267***	***0.013***	0.153	0.160	0.073	0.504
Interval since TBI (month)	− 0.001	0.993	− 0.081	0.457	− 0.181	0.096	− 0.187	0.085
GCS	− 0.069	0.561	− 0.056	0.641	*** − 0.286***	***0.014***	− 0.216	0.066

Data were expressed as correlation coefficient and *P*-values. Significant correlations are highlighted in bold and italic numbers

As both TMT-A and TMT-B were not normally distributed, Spearman’s rank correlation test was used

*MMSE*, Mini-Mental State Examination; *CDT*, Clock Drawing Test; *TMT*, Trail Making Test; *GCS*, Glasgow Coma Scale

**Table 2:** Correlations between values of MMSE, CDT, TMT-A, and TMT-B and values of bio-signals and time-domain cardiac autonomic parameters in 86 patients with a history of traumatic brain injury

	MMSE		CDT		TMT-A (s)		TMT-B (s)	
	Rho	*P*	Rho	*P*	Rho	*P*	Rho	*P*
RRI (ms)	0.198	0.071	0.167	0.129	*** − 0.296***	***0.006***	− 0.210	0.055
RESP (cpm)	− 0.116	0.295	0.065	0.558	0.089	0.420	0.144	0.357
BPsys (mmHg)	0.121	0.274	0.100	0.366	− 0.024	0.830	− 0.029	0.562
BPdia (mmHg)	0.050	0.274	0.101	0.361	0.128	0.247	− 0.024	0.826
RRI-CV (%)	0.100	0.364	− 0.035	0.752	− 0.139	0.208	− 0.040	0.719
RRI-SD (ms)	0.139	0.208	0.040	0.717	− 0.195	0.076	− 0.082	0.459
RMSSD (ms)	0.131	0.236	0.091	0.411	− 0.210	0.055	− 0.124	0.261

Data are showing the correlation coefficients and *P*-values. Significant correlations are highlighted in bold and italic numbers

As both TMT-A and TMT-B were not normally distributed, Spearman’s rank correlation was used

*MMSE*, Mini-Mental State Examination; *CDT*, Clock Drawing Test; *TMT*, Trail Making Test; *RRI*, RR interval; *RESP*, respiratory frequency; *BPsys*, systolic blood pressure; *BPdia*, diastolic blood pressure; *CV*, coefficient of variation; *SD*, standard deviation; *RMSSD*, square root of mean squared differences of successive RR intervals

**Table 3:** Correlations between values of MMSE, CDT, TMT-A, and TMT-B and values of frequency-domain cardiovascular autonomic parameters in 86 patients with a history of traumatic brain injury

	MMSE		CDT		TMT-A (s)		TMT-B (s)	
	Rho	*P*	Rho	*P*	Rho	*P*	Rho	*P*
RRI-LF-powers (ms^2^)	0.120	0.277	0.023	0.836	− 0.176	0.110	− 0.017	0.881
RRI-HF-powers (ms^2^)	0.102	0.355	0.019	0.863	*** − 0.233***	***0.033***	− 0.172	0.117
RRI-total-powers (ms^2^)	0.143	0.194	0.028	0.803	− 0.210	0.055	− 0.116	0.292
RRI-LFnu-powers (%)	0.078	0.479	0.099	0.368	0.203	0.064	***0.265***	***0.015***
RRI-HFnu-powers (%)	− 0.078	0.479	− 0.099	0.368	− 0.203	0.064	*** − 0.265***	***0.015***
RRI-LF/HF ratio	0.073	0.509	0.102	0.355	0.192	0.081	***0.230***	***0.036***
BPsys-LF-powers (mmHg^2^)	− 0.080	0.469	− 0.075	0.500	0.040	0.720	***0.236***	***0.030***
BPsys-HF-powers (mmHg^2^)	0.040	0.719	− 0.231	0.355	− 0.046	0.675	− 0.205	0.061
BRS (ms/mmHg)	0.117	0.290	0.125	0.256	*** − 0.221***	***0.043***	− 0.147	0.181

Data are showing the correlation coefficients and *P*-values. Significant correlations are highlighted in bold and italic numbers

As both TMT-A and TMT-B were not normally distributed, Spearman’s rank correlation was used

*MMSE*, Mini-Mental State Examination; *CDT*, Clock Drawing Test; *TMT*, Trail Making Test; *RRI*, RR intervals; *BPsys*, systolic blood pressure; *LF*, low frequency; *HF*, high frequency; *nu*, normalized unit; *BRS*, baroreflex sensitivity

Values of the Clock Drawing Test only correlated with the patients’ age (Spearman’s rho = 0.267, *P* = 0.013, Table 1) but did not correlate significantly with other demographic data, bio-signals, or time- and frequency-domain autonomic parameters (Tables 1, 2, and 3).

**Fig. 1:**
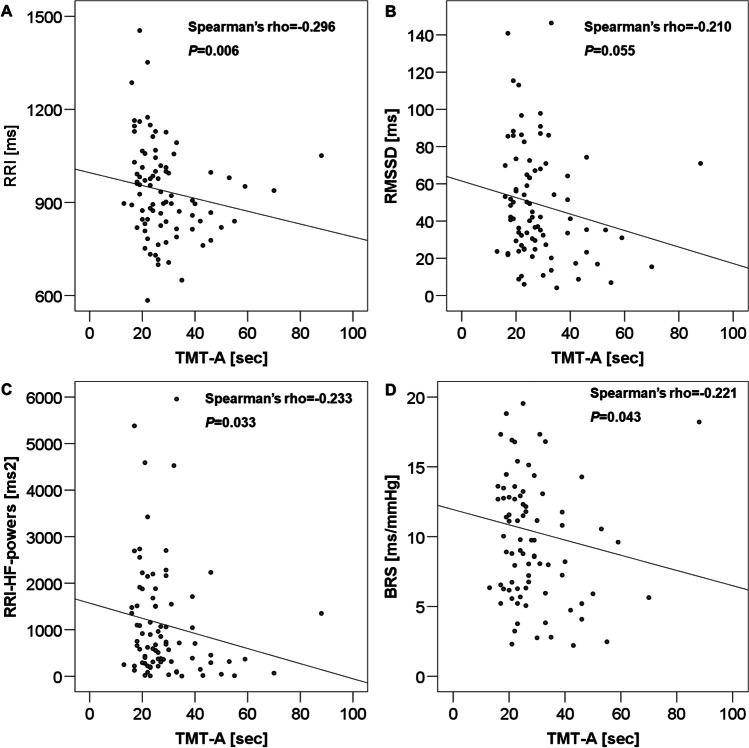
Correlations between values of TMT-A and RRI (panel **A**), RMSSD (panel **B**), RRI-HF-powers (panel **C**), and BRS (panel **D**). TMT-A inversely correlated with RRI, RMSSD, RRI-HF-powers, and BRS. TMT, Trail Making Test; RRI, RR intervals; RMSSD, square root of mean squared differences of successive RR intervals; HF, high frequency; BRS, baroreflex sensitivity

TMT-A values correlated negatively with the GCS scores assessed at the time of the initial trauma (Spearman’s rho =  − 0.286, *P* = 0.014) and with RRIs (Spearman’s rho =  − 0.296, *P* = 0.006) as well as RRI-HF powers (Spearman’s rho =  − 0.233, *P* = 0.033; Fig. 1, Table 3) and BRS (Spearman’s rho = 0.221, *P* = 0.043; Fig. 1, Table 3) while the correlation with RMSSD values was not quite significant (Spearman’s rho =  − 0.210, *P* = 0.055).

However, TMT-A values did not correlate with the patients’ age and the duration of the interval since the trauma (Table 1), nor with BPsys, BPdia, respiration, and other autonomic parameters (Tables 2 and 3).

**Fig. 2:**
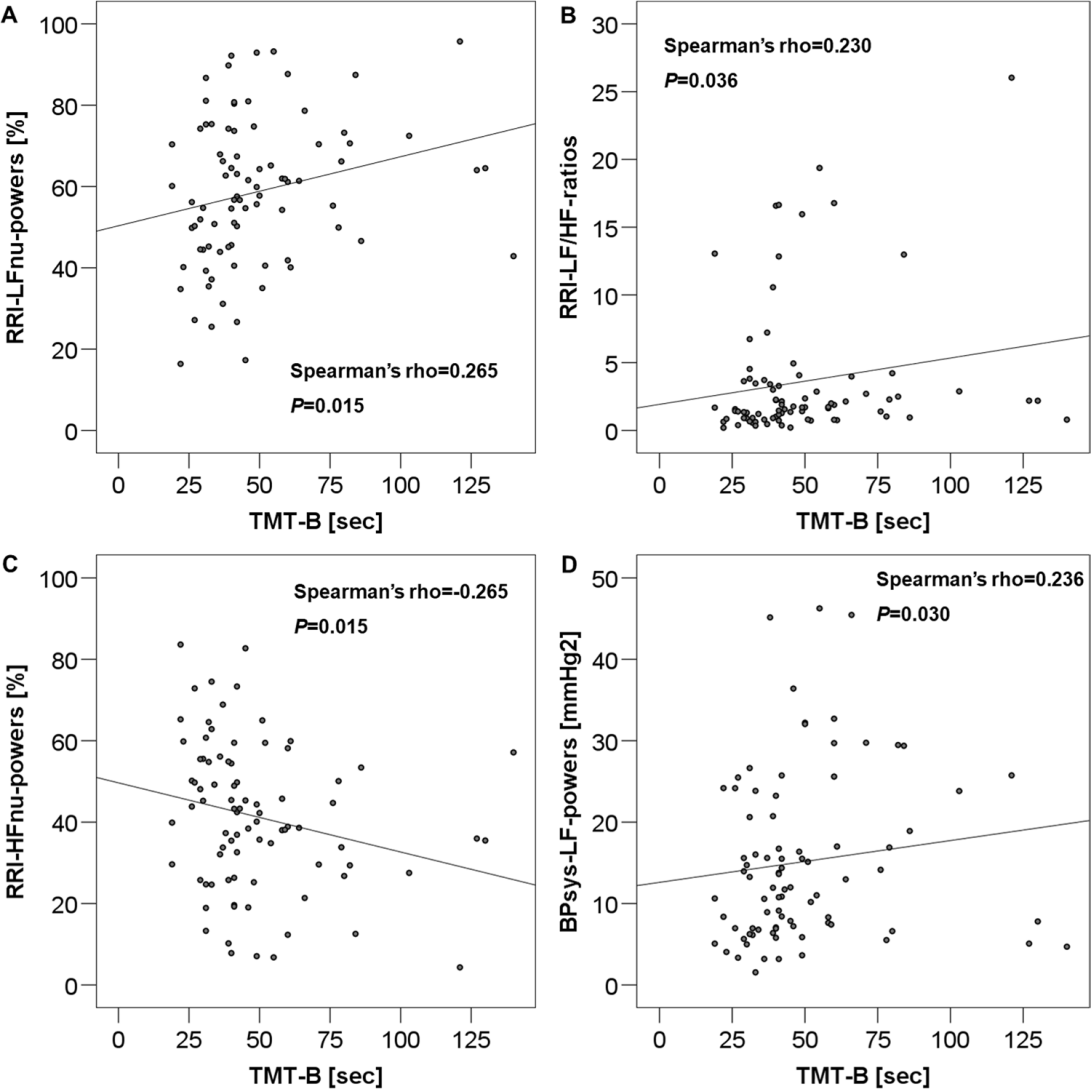
Correlations between values of TMT-B and RRI-LFnu-powers (panel **A**), RRI-LF/HF-ratios (panel **B**), RRI-HFnu-powers (panel **C**), and BPsys-LF-powers (panel **D**). TMT-B positively correlated with RRI-LFnu-powers, RRI-LF/HF-ratios, and BPsys-LF-powers, but negatively with RRI-HFnu-powers. TMT, Trail Making Test; RRI, RR intervals; HF, high frequency; LF, low frequency; nu, normalized unit; BPsys, systolic blood pressure

TMT-B values positively correlated with RRI-LFnu-powers (Spearman’s rho = 0.265, *P* = 0.015; Fig. 2, Table 3), RRI-LF/HF-ratios (Spearman’s rho = 0.230, *P* = 0.036; Fig. 2, Table 3), and BPsys-LF-powers (Spearman’s rho = 0.236, *P* = 0.030; Fig. 2, Table 3), and negatively correlated with RRI-HFnu-powers (Spearman’s rho =  − 0.265, *P* = 0.015; Fig. 2, Table 3). However, the correlation between TMT-B values and RRIs was not quite significant (Spearman’s rho =  − 0.210, *P* = 0.055).

Yet, the correlation between TMT-B values and the GCS scores assessed at the time of the initial trauma was not quite significant (Spearman’s rho =  − 0.216, *P* = 0.066).

TMT-B values also did not correlate with the patient’s age and the duration of the interval since the trauma (Table 1), nor with BPsys, BPdia, Resp, and other autonomic parameters (Tables 2 and 3).

There was no significant difference in autonomic parameters nor in the results of the applied cognitive function tests between our 43 patients with a history of mild TBI and our 43 patients with a history of moderate or severe TBI (Supplementary Tables 1 and 2).

The gender-specific analysis showed that bio-signals and cardiovascular autonomic parameters also did not differ between the 64 male and 22 female post-TBI-patients except for higher values of sympathetically mediated LF-powers of BP modulation (Supplementary Table 1). Moreover, the values of MMSE, CDT, TMT-A, and TMT-B did not differ between male and female post-TBI-patients (Supplementary Table 2).

However, in the group of the 64 male post-TBI-patients, TMT-A values correlated negatively with RRI (Spearman’s rho =  − 0.380, *P* = 0.002), RRI-HF-powers (Spearman’s rho =  − 0.264, *P* = 0.038), and RRI-HF-nu-powers (Spearman’s rho =  − 0.333, *P* = 0.008), but positively with RRI-LF-nu-powers (Spearman’s rho = 0.333, *P* = 0.008) and RRI-LF/HF-ratios (Spearman’s rho = 0.323, *P* = 0.010); TMT-B values correlated negatively with RRI-HFnu-powers (Spearman’s rho =  − 0.329, *P* = 0.009) and positively with RRI-LFnu-powers (Spearman’s rho = 0.329, *P* = 0.009), RRI-LF/HF-ratio (Spearman’s rho = 0.299, *P* = 0.018), and BPsys-LF-powers (Spearman’s rho = 0.273, *P* = 0.032). In contrast, in the 22 female post-TBI-patients, TMT-A values and TMT-B values did not correlate significantly with the bio-signals and autonomic parameters (Supplementary Tables 3 to 6).

## Discussion

Our data show — to our knowledge, for the first time — that there are associations between the impairment of cardiovascular autonomic regulation and altered cognitive performance in patients with a history of TBI. However, only selected cognitive tests and autonomic parameters correlated with each other. The MMSE that is widely used as a clinical screening test of cognitive impairment [20, 26] showed no abnormal results in our patients and thus also could not reveal any association with cardiovascular autonomic dysregulation. In contrast, there was a correlation between a decrease in visuospatial abilities at the time of our evaluation, as assessed by the part A of the TMT, and the severity of the initial TBI, as assessed by the GCS scores at the time of the injury (Table 1). Furthermore, lower visuospatial performance correlated with reduced parasympathetic function as evidenced by lower RRIs, i.e., higher heart rates, reduced parasympathetically mediated RRI-HF-powers and lower BRS (Fig. 1, Table 3) in patients with lower TMT-A scores.

Moreover, our data confirmed the association of a shift in sympathetic-parasympathetic cardiovascular balance towards sympathetic predominance with reduced parasympathetic control in patients with impairment of executive function: poorer executive function, as reflected by higher TMT-B scores correlated with sympathetically mediated, normalized RRI-LF-powers, with RRI-LF/HF-ratios, the index of sympathetic-parasympathetic balance, and with the sympathetically mediated powers of BP modulation (Fig. 2, Table 3), while higher TMT-B scores were associated with lower parasympathetically cardiac modulation, measured as RRI-HFnu-powers (Fig. 2, Table 3).

The negative correlation between TMT-A scores and GCS scores and the trend towards a negative correlation (*P* = 0.066) between higher TMT-B scores and lower GCS scores suggest that patients with a history of a more severe TBI have a more prominent decrease in visuospatial function and at least a trend towards a more prominent decrease in executive function. The study by Demery et al. supports our conclusions: using the TMT-A and TMT-B, the authors compared visuospatial and executive functions between 20 patients with a history of mild TBI, 26 patients with a history of (moderate to) severe TBI, and 24 (age- and sex-matched) healthy controls and found a stepwise deterioration of TMT-A scores and TMT-B scores in patients with mild and patients with (moderate to) severe TBI compared to the scores of the controls [22]. In another study with 67 TBI patients, the authors found that patients with a higher GCS score, i.e., with a less severe TBI, had better scores on the Montreal Cognitive Assessment-Basic, i.e., a better cognitive function [27]. In a recent study of 15,764 persons aged 50 to 90 years, Lennon et al. moreover demonstrated a cumulative effect of TBIs on cognition, particularly on attention and executive function, even decades after the TBIs [29]. The authors showed that a higher number of experienced mild TBIs is associated with a worse cognitive performance, particularly poorer attention and executive function, even decades after the trauma [29].

While there seem to be no studies that assessed associations between impaired cardiovascular autonomic function and altered cognitive function in patients with a history of mild TBI, there are various studies in patients with other neurological or neuropsychiatric disorders that demonstrated an association between cognitive performance and cardiac autonomic regulation [9, 10, 14].

Struhal and colleagues tested autonomic function in 26 patients with the behavioral variant of frontotemporal dementia (bvFTD) and in 18 patients with Alzheimer’s disease (AD) [12, 13]. Using the Ewing battery, the authors found cardiovascular autonomic dysregulation in 42% of their bvFTD patients and in 44% of their AD patients and assumed that insular involvement in the neurodegenerative process may have contributed to the autonomic dysregulation in both patient groups [12, 13]. In a meta-analysis comprising 27 studies of patients with different neurodegenerative disorders (including mild cognitive impairment in 9 studies, Alzheimer disease in 7 studies, vascular dementia in 5 studies, frontotemporal dementia in 2 studies, Parkinson’s disease in 6 studies, Lewy body dementia in 1 study, Huntington’s disease in 1 study, and multiple sclerosis in 1 study), Liu et al. found an association between poorer cognitive scores and a reduced cardiovagal modulation [29], i.e., results similar to the associations seen in our patients. The group concluded that parasympathetically mediated parameters of heart rate modulation are linked to cognitive and behavioral function in patients with neurodegenerative diseases [29]. This conclusion is supported by the results of a study by Kim et al. [9]. In 311 community-dwelling women above the age of 64 who had difficulties in two or more physical domains of disability and MMSE scores above 17, Kim and co-workers found that reduced parasympathetic cardiac modulation was associated with an almost seven times greater odds ratio of cognitive impairment, defined as MMSE scores below 24 [9]. In 3583 older participants with a mean age of 75 years considered at risk of cardiovascular disease, Mahinrad performed cognitive testing and found that participants with reduced RRI-SD values, i.e., lowered sympathetic and parasympathetic cardiac modulation, had impaired reaction times and processing speed, i.e., impaired cognitive function [10]. In 30 patients diagnosed with chronic fatigue syndrome, Beaumont et al. reported an association between reduced cardiovagal tone, as assessed by RMSSD, and cognitive impairment evaluated by the digit symbol test, the spatial working memory, and the Stroop task [14]. Similarly, Martin et al. evaluated 916 healthy older persons and found a close association between reduced cardiac BRS and an increased risk of memory impairment [29]. Moreover, Santos et al. showed that increased sympathetic cardiac activation, reflected by increased RRI-LF/HF-ratios, upon orthostatic challenge correlated with lower executive function scores among 62 community-dwelling normotensive and hypertensive subjects [11].

While these studies support our conclusion that there is an association between cardiac autonomic dysfunction and cognitive impairment in patients with brain lesions, various neuroimaging studies tested which brain regions contribute to cognitive processing and whether these regions overlap with areas of the central autonomic network lend further support to our conclusion [31–34].

For example, Thayer et al. conclude from their and other studies [15, 16, 31, 32, 34] that there is an overlap between brain areas and networks involved in the central autonomic cardiac modulation and structures associated with cognitive regulation [16]. Areas of the autonomic network, particularly the prefrontal cortex regions, but also the anterior cingulate cortex, or the amygdala, are involved in autonomic control and modulation [33, 35] but also contribute to cognitive tasks, especially to executive performance [15, 31]. The prefrontal cortex directly or indirectly interacts with many other structures of the central autonomic network, such as the insula, hypothalamus, periaqueductal gray, parabrachial pontine nuclei as well as the nucleus of the solitary tract, and the rostral and caudal ventrolateral medulla, and ultimately modifies sympathetic and parasympathetic outflow [15, 31].

After TBI, central autonomic network structures such as the prefrontal cortex, anterior cingulate cortex, or amygdala might be afflicted by the trauma. In previous studies of patients with a history of TBI, we found CAD months and years after the injury not only in moderate-severe TBI but also after mild TBI [3, 5, 7]. Conventional neuroimaging methods using computed tomography or magnetic resonance imaging (MRI) show macroscopic brain lesions only in moderate-severe TBI patients but not in mild TBI patients [18]. However, even in patients with mild TBI, refined imaging techniques such as voxel-based volume analysis and diffusion tensor weighted MRIs, or functional MRIs, revealed structural and functional abnormalities in various brain regions including, e.g., the prefrontal cortex, cingulate region, and white matter regions [36]. Therefore, and based on the aforementioned common pathways of autonomic modulation and cognitive processing, we assume that the rather mild cognitive impairment and cardiovascular autonomic alterations recorded in our post-TBI-patients can be ascribed to trauma induced lesions of structures involved in both central autonomic modulation and cognition.

Our results showed no major differences in autonomic parameters between the 64 male and 22 female post-TBI-patients. Only sympathetically mediated BP modulation — reflected by BP-LF-powers — was higher among the male than the female patients (Supplementary Table 1). The finding is consistent with the fact that sympathetic modulation may be higher in men than in women already under resting conditions [37]. The relative sympathetic predominance among male post-TBI-patients is to some extent supported by a study of Pyndiura and co-workers in female and male athletes with or without a history of mild TBI [38]. The authors found higher heart rates in male athletes with a history of concussion than in male athletes without a history of concussion, while they found no such differences in female athletes with or without a history of concussion; similar to our results, the authors found no gender differences in other parameters of heart rate variability [38].

As expected, results of cognitive function were similar between male and female post-TBI-patients; however, only in our 64 male patients did we see correlations between impaired executive and visuospatial functions, as expressed by TMT-A and TMT-B values, and reduced parameters of parasympathetic but increased parameters of sympathetic cardiovascular modulation (Tables 2 and 3; Figs. 1 and 2). The lack of such correlations among the female patients was probably due to the rather limited sample size of only 22 female post-TBI-patients.

Somewhat unexpectedly, we also found no significant differences in autonomic parameters nor in test results of cognitive function between the patients with a history of mild TBI and those with a history of moderate or severe TBI. This lack of differences in autonomic or cognitive parameters between the two patient groups might have been due to the fact that we only enrolled patients who — at the time of our evaluation, i.e., after a median interval of 25.5 months between the initial TBI and our evaluation — had no clinically overt neurological or autonomic dysfunction and did not complain about any cognitive difficulties but pursued their daily and professional tasks without manifest post-traumatic limitations. Moreover, our results show that autonomic and cognitive changes were rather mild in all our post-TBI-patients (Supplementary Tables 1–2). Most likely, these minor changes account for the lack of significant differences in the results between our patients with a history of mild TBI and our patients with a history of moderate-severe TBI.

### Limitations

Our study has several limitations. Firstly, the sample size of our TBI patients was relatively small due to the careful inclusion of patients with a history of TBI and exclusion of any patients with any additional disease or on any medication possibly afflicting autonomic modulation. A larger sample size might have unveiled further associations between parameters indicating cognitive impairment and parameters of autonomic dysfunction. Secondly, none of the post-TBI-patients had MMSE score below 24, i.e., scores indicating cognitive impairment. Perhaps, a more sensitive cognitive testing battery such as the Montreal Cognitive Assessment [39] might detect subtle global cognitive impairment even in patients who had a mild TBI in the past.

## Conclusions

In summary, our study confirmed that there was an association between altered cognitive function and cardiovascular autonomic modulation in our patients who had suffered a mild or moderate-severe TBI in the past 25.5 months (interquartile range 14–55 months) and that there is an association between the severity of cognitive-executive impairment and the decrease in parasympathetic cardiac modulation, the concomitant shift towards sympathetic cardiovascular predominance, and the reduced baroreflex sensitivity (Tables 2 and 3, Figs. 1 and 2). CAD is known to be associated with an unfavorable clinical prognosis and increased long-term cardiovascular risk [3–7]. The finding of concomitant cognitive changes further aggravates the patients’ quality of life and possibly prognosis [40]. Thus, both autonomic function and cognition should be monitored in post-TBI-patients.

## Supplementary Information

Below is the link to the electronic supplementary material.

Supplementary file1 (DOCX 42 KB)

## Data Availability

Data supporting the results reported in the article are available upon reasonable request to the corresponding author.
