# Supplementary material for: Cardiovascular autonomic regulation correlates with cognitive performance in patients with a history of traumatic brain injury
Source: Neurol Sci. 2023 May 25;44(10):3535–44. doi: 10.1007/s10072-023-06857-y (PMC10495484; doi:10.1007/s10072-023-06857-y)
Supplement: Supplementary file 1 — Supplementary file1 (DOCX 42 KB) [file 10072_2023_6857_MOESM1_ESM.docx]

Supplementary table 1. Comparisons of bio-signals and autonomic parameters between subgroups of patients with a history of traumatic brain injury (TBI).

| Parameters | Male post-TBI patients  n=64  [I] | Female post-TBI patients  n=22  [II] | P-Value  I v. II | Mild post-TBI patients  n=43  [III] | Moderate or severe post-TBI patients  n=43  [IV] | P-Value  III vs. IV |
| --- | --- | --- | --- | --- | --- | --- |
|  |  |  |  |  |  |  |
| RRI [ms] | 954.0±167.3 | 888.0±90.0 | 0.083 | 909.1±141.0 | 964.3±161.6 | 0.099 |
| RESP [cpm] | 14.7±3.0 | 15.4±3.6 | 0.481 | 14.9±4.4 | 14.9±3.4 | 0.923 |
| BPsys [mmHg] | 126.1±17.2 | 120.4±14.8 | 0.177 | 125.1±17.8 | 124.0±15.7 | 0.763 |
| BPdia [mmHg] | 64.5±10.3 | 63.8±10.2 | 0.784 | 65.4±10.5 | 63.2±9.9 | 0.314 |
| RRI-CV [%] | 5.5±2.6 | 4.7±2.1 | 0.222 | 5.1±2.6 | 5.5±2.3 | 0.471 |
| RRI-SD [ms] | 52.9±27.6 | 42.9±21.4 | 0.112 | 47.5±28.7 | 53.0±23.7 | 0.334 |
| RMSSD [ms] | 51.0±31.5 | 42.6±25.6 | 0.259 | 44.8±33.2 | 52.8±26.6 | 0.228 |
| RRI-LF-powers [ms^2^] | 1919.1±2353.2 | 1080.5±1151.7 | 0.130 | 1634.9±2417.1 | 1763.9±1829.2 | 0.248 |
| RRI-HF-powers [ms^2^] | 1175.2±1253.9 | 913.0±1097.2 | 0.360 | 1035.9±1371.6 | 1177.1±1044.9 | 0.122 |
| RRI-total-powers [ms^2^] | 3094.3±3264.6 | 1993.5±2118.8 | 0.086 | 2670.9±3575.2 | 2941.1±2411.8 | 0.171 |
| RRI-LFnu-powers [%] | 60.1±19.6 | 54.7±12.2 | 0.230 | 60.3±18.4 | 57.1±17.8 | 0.419 |
| RRI-HFnu-powers [%] | 39.9±19.6 | 45.3±12.2 | 0.230 | 39.7±18.4 | 42.9±17.8 | 0.419 |
| RRI-LF/HF-ratio | 4.2±5.6 | 1.8±1.4 | 0.176 | 4.6±6.3 | 2.6±3.0 | 0.629 |
| BPsys-LF-powers [mmHg^2^] | **16.6±10.9** | **11.1±7.4** | **0.033** | 16.6±11.3 | 13.6±9.3 | 0.376 |
| BPsys-HF-powers [mmHg^2^] | 4.2±3.3 | 3.8±3.1 | 0.515 | 4.1±3.4 | 4.1±3.0 | 0.936 |
| BRS [ms/mmHg] | 10.3±4.9 | 10.6±6.9 | 0.583 | 9.5±5.3 | 11.3±5.7 | 0.140 |

RRI: RR-interval; RESP: respiratory frequency; BPsys: systolic blood pressure; BPdia: diastolic blood pressure; CV: coefficient of variation; SD: standard deviation; RMSSD: square root of mean squared differences of successive RR intervals; LF: low frequency; HF: high frequency; nu: normalized unit; BRS: baroreflex sensitivity.

Among the healthy controls, autonomic parameters did not differ between women and men. Among TBI patients, BPsys-LF-powers were significantly lower in TBI women than in TBI men.

Supplementary table 2. Comparisons of cognitive parameters between subgroups of patients with a history of traumatic brain injury (TBI).

| Parameters | Male post-TBI patients  n=64  [I] | Female post-TBI patients  n=22  [II] | P-Value  I vs. II | Mild post-TBI patients  n=43  [III] | Moderate or severe post-TBI patients  n=43  [IV] | P-Value  III vs. IV |
| --- | --- | --- | --- | --- | --- | --- |
|  |  |  |  |  |  |  |
| Age (years) | 31.9 ± 10.6 | 36.7 ± 10.0 | 0.074 | 34.3 ± 12.1 | 32.0 ± 9.5 | 0.476 |
| MMSE | 29.4 ± 1.0 | 29.2 ± 1.1 | 0.454 | 29.4 ± 1.0 | 29.4 ± 1.2 | 0.478 |
| CDT | 1.3 ± 0.5 | 1.2 ± 0.5 | 0.627 | 1.3 ± 0.6 | 1.3 ± 0.5 | 0.534 |
| TMT-A (s) | 28.0 ± 12.3 | 29.7 ± 12.1 | 0.510 | 26.4 ± 9.9 | 30.3 ± 14.3 | 0.100 |
| TMT-B (s) | 46.4 ± 21.4 | 56.2 ± 31.5 | 0.229 | 44.3 ± 21.3 | 53.4 ± 26.9 | 0.076 |

MMSE: Mini-Mental State Examination; CDT: clock drawing test; TMT: Trail Making Test.

Supplementary table 3. Correlations between values of MMSE, CDT, TMT-A, TMT-B and values of bio-signals and time-domain cardiac autonomic parameters in 64 men with a history of traumatic brain injury

|  | MMSE | | CDT | | TMT-A [s] | | TMT-B [s] | |
| --- | --- | --- | --- | --- | --- | --- | --- | --- |
|  | Rho | P | Rho | P | Rho | P | Rho | P |
| RRI [ms] | 0.266 | 0.036 | 0.055 | 0.671 | **-0.380** | **0.002** | -0.236 | 0.064 |
| RESP [cpm] | -0.206 | 0.109 | -0.049 | 0.703 | 0.045 | 0.731 | -0.200 | 0.119 |
| BPsys [mmHg] | 0.026 | 0.839 | 0.182 | 0.157 | -0.101 | 0.435 | -0.272 | 0.033 |
| BPdia [mmHg] | -0.031 | 0.813 | 0.147 | 0.255 | 0.069 | 0.593 | -0.065 | 0.617 |
| RRI-CV [%] | 0.190 | 0.139 | -0.094 | 0.470 | -0.073 | 0.571 | 0.037 | 0.778 |
| RRI-SD [ms] | 0.252 | 0.048 | -0.022 | 0.862 | -0.169 | 0.190 | -0.031 | 0.811 |
| RMSSD [ms] | 0.240 | 0.060 | 0.014 | 0.916 | -0.245 | 0.055 | -0.094 | 0.466 |

Data are showing the correlation coefficients and *P*-values. Significant correlations are highlighted in bold numbers. As both TMT-A and TMT-B were not normally distributed, Spearman’s rank correlation was used. MMSE: Mini-Mental State Examination; CDT: clock drawing test; TMT: Trail Making Test; RRI: RR-interval; RESP: respiratory frequency; BPsys: systolic blood pressure; BPdia: diastolic blood pressure; CV: coefficient of variation; SD: standard deviation; RMSSD: square root of mean squared differences of successive RR intervals.

Supplementary table 4. Correlations between values of MMSE, CDT, TMT-A, TMT-B and values of frequency-domain cardiovascular autonomic parameters in 64 men with a history of traumatic brain injury

|  | | MMSE | | | CDT | | TMT-A [s] | | TMT-B [s] | |
| --- | --- | --- | --- | --- | --- | --- | --- | --- | --- | --- |
|  |  | Rho | | P | Rho | P | Rho | P | Rho | P |
| RRI-LF-powers [ms^2^] | | 0.206 | 0.109 | -0.013 | 0.921 | -0.098 | 0.448 | 0.053 | 0.684 |  |
| RRI-HF-powers [ms^2^] | | 0.220 | 0.086 | -0.091 | 0.482 | **-0.264** | **0.038** | -0.171 | 0.185 |  |
| RRI-total-powers [ms^2^] | | 0.259 | 0.042 | -0.057 | 0.658 | -0.190 | 0.140 | -0.078 | 0.549 |  |
| RRI-LFnu-powers [%] | | 0.032 | 0.804 | 0.199 | 0.120 | **0.333** | **0.008** | **0.329** | **0.009** |  |
| RRI-HFnu-powers [%] | | -0.032 | 0.804 | -0.199 | 0.120 | **-0.333** | **0.008** | **-0.329** | **0.009** |  |
| RRI-LF/HF-ratio | | 0.024 | 0.853 | 0.194 | 0.130 | **0.323** | **0.010** | **0.299** | **0.018** |  |
| BPsys-LF-powers [mmHg^2^] | | -0.168 | 0.193 | -0.020 | 0.876 | 0.188 | 0.143 | **0.273** | **0.032** |  |
| BPsys-HF-powers [mmHg^2^] | | 0.007 | 0.954 | -0.170 | 0.186 | -0.040 | 0.760 | -0.234 | 0.067 |  |
| BRS [ms/mmHg] | | 0.276 | 0.030 | 0.013 | 0.922 | -0.232 | 0.069 | -0.120 | 0.352 |  |

Data are showing the correlation coefficients and *P*-values. Significant correlations are highlighted in bold numbers. As both TMT-A and TMT-B were not normally distributed, Spearman’s rank correlation was used. MMSE: Mini-Mental State Examination; CDT: clock drawing test; TMT: Trail Making Test; RRI: RR-intervals; BPsys: systolic blood pressure; LF: low frequency; HF: high frequency; nu: normalized unit; BRS: baroreflex sensitivity.

Supplementary table 5. Correlations between values of MMSE, CDT, TMT-A, TMT-B and values of bio-signals and time-domain cardiac autonomic parameters in 22 women with a history of traumatic brain injury

|  | MMSE | | CDT | | TMT-A [s] | | TMT-B [s] | |
| --- | --- | --- | --- | --- | --- | --- | --- | --- |
|  | Rho | P | Rho | P | Rho | P | Rho | P |
| RRI [ms] | -0.052 | 0.820 | -0.014 | 0.952 | -0.052 | 0.820 | 0.100 | 0.658 |
| RESP [cpm] | 0.244 | 0.274 | 0.035 | 0.876 | 0.244 | 0.274 | -0.014 | 0.950 |
| BPsys [mmHg] | 0.129 | 0.567 | -0.258 | 0.246 | 0.129 | 0.567 | -0.029 | 0.899 |
| BPdia [mmHg] | 0.388 | 0.074 | -0.013 | 0.956 | 0.388 | 0.074 | 0.134 | 0.552 |
| RRI-CV [%] | -0.294 | 0.183 | -0.093 | 0.682 | -0.294 | 0.183 | -0.211 | 0.345 |
| RRI-SD [ms] | 0.271 | 0.222 | 0.167 | 0.459 | -0.271 | 0.222 | -0.179 | 0.427 |
| RMSSD [ms] | -0.102 | 0.652 | 0.332 | 0.131 | -0.102 | 0.652 | -0.142 | 0.527 |

Data are showing the correlation coefficients and *P*-values. Significant correlations are highlighted in bold numbers.

As both TMT-A and TMT-B were not normally distributed, Spearman’s rank correlation was used. MMSE: Mini-Mental State Examination; CDT: clock drawing test; TMT: Trail Making Test; RRI: RR-interval; RESP: respiratory frequency; BPsys: systolic blood pressure; BPdia: diastolic blood pressure; CV: coefficient of variation; SD: standard deviation; RMSSD: square root of mean squared differences of successive RR intervals.

Supplementary table 6. Correlations between values of MMSE, CDT, TMT-A, TMT-B and values of frequency-domain cardiovascular autonomic parameters in 22 women with a history of traumatic brain injury

|  | | MMSE | | | CDT | | TMT-A [s] | | TMT-B [s] | |
| --- | --- | --- | --- | --- | --- | --- | --- | --- | --- | --- |
|  |  | Rho | | P | Rho | P | Rho | P | Rho | P |
| RRI-LF-powers [ms^2^] | | -0.075 | 0.140 | 0.118 | 0.602 | -0.387 | 0.075 | -0.189 | 0.400 |  |
| RRI-HF-powers [ms^2^] | | -0.173 | 0.442 | 0.315 | 0.154 | -0.156 | 0.487 | -0.1531 | 0.496 |  |
| RRI-total-powers [ms^2^] | | -0.110 | 0.628 | 0.202 | 0.368 | -0.262 | 0.239 | -0.185 | 0.409 |  |
| RRI-LFnu-powers [%] | | 0.167 | 0.457 | -0.318 | 0.149 | -0.255 | 0.251 | 0.108 | 0.633 |  |
| RRI-HFnu-powers [%] | | -0.167 | 0.457 | 0.318 | 0.149 | 0.255 | 0.251 | -0.108 | 0.633 |  |
| RRI-LF/HF-ratio | | 0.179 | 0.426 | -0.336 | 0.127 | -0.241 | 0.281 | 0.072 | 0.751 |  |
| BPsys-LF-powers [mmHg^2^] | | 0.081 | 0.721 | -0.300 | 0.174 | -0.294 | 0.184 | 0.332 | 0.131 |  |
| BPsys-HF-powers [mmHg^2^] | | 0.086 | 0.702 | -0.406 | 0.061 | -0.082 | 0.718 | -0.124 | 0.582 |  |
| BRS [ms/mmHg] | | -0.340 | 0.122 | 0.243 | 0.277 | -0.139 | 0.536 | -0.130 | 0.564 |  |

Data are showing the correlation coefficients and *P*-values. Significant correlations are highlighted in bold numbers. As both TMT-A and TMT-B were not normally distributed, Spearman’s rank correlation was used. MMSE: Mini-Mental State Examination; CDT: clock drawing test; TMT: Trail Making Test; RRI: RR-intervals; BPsys: systolic blood pressure; LF: low frequency; HF: high frequency; nu: normalized unit; BRS: baroreflex sensitivity.
